# Unique, Not Psychogenic Movements: Painful Leg and Moving Toes Syndrome

**DOI:** 10.7759/cureus.29169

**Published:** 2022-09-14

**Authors:** Shuichiro Neshige, Megumi Nonaka

**Affiliations:** 1 Department of Clinical Neuroscience and Therapeutics, Hiroshima University, Hiroshima, JPN

**Keywords:** acute pain, interested in clinical neurology, lumbar spondylosis, elderly individuals, involuntary movement

## Abstract

A 74-year-old woman visited our department for distally predominant unpleasant pain in the bilateral feet for several months. She had a history of chronic lumbago. Neurological examinations showed normal findings other than involuntary movements. A nerve conduction study, electroencephalography, and brain MRI revealed unremarkable findings, while spinal MRI revealed mild lumbar spinal stenosis. Given the typical unique movements, i.e., bilateral toe movements, which are asynchronous and consist of extension, flexion, and, rarely, abduction, she was diagnosed with painful leg moving toes syndrome. Administration of duloxetine produced partial pain relief and reduced movements. We considered that clinicians should be aware of this unique movement disorder in order to avoid misdiagnosis with psychogenic conditions.

## Introduction

Painful legs and moving toes (PLMT) is a rare movement condition defined by pain in one or more limbs and accompanied by repetitive, non-rhythmic finger motions [1]. It was firstly reported by Spillane et al. as a disease associated with lower extremity pain and involuntary toe movements in 1971 [2]. The precise pathophysiologic mechanism is unknown [3]. Pain severity varies greatly among individuals, ranging from persistent discomfort to severe pain. Additionally, some patients had no identifiable cause for the pain. Thus, clinicians might make the movements for a psychological condition. Here, we report an elderly woman who was diagnosed with painful leg moving toes syndrome. Video shows typical unique movements, i.e., bilateral toe movements, which are asynchronous and consist of extension, flexion, and, rarely, abduction. Multiple neurological examinations showed unremarkable findings.

## Case presentation

A 74-year-old woman with a history of chronic lumbago presented with distally predominant unpleasant pain in the bilateral feet for several months. Neurological examination, manual muscle test, muscle tonus, and deep tendon reflex showed normal findings. Straight leg raising and femoral nerve stretching tests were unremarkable. The sensory disturbance was negligible except for pain during spontaneous toe movements, i.e., spontaneous bilateral toe movements, which were asynchronous and worse on the left, were visible (Video 1).

**Video 1:** Involuntary movements in the bilateral toes The movements consist of extension, flexion, and, rarely, abduction of the toes.

The movements consisted of extension, flexion, and, rarely, abduction of the toes, which was often visible during a resting condition, including during sleep. Additionally, the movements were not inhibited by several tasks, including eye closure, calculation, and auditory stimuli. A nerve conduction study showed unremarkable findings. Electroencephalography showed a well-organized posterior dominant rhythm, 9 to 10 Hz. There were no epileptic discharges or slows. A needle electromyogram showed no denervation. While brain MRI revealed normal findings, spinal MRI revealed mild lumbar spinal stenosis. There were no abnormal signals on the dorsal root nerve. Work-up for malignancy showed negative findings. Hence, we diagnosed the patient with painful legs and moving toes syndrome (PLMT). Oral diazepam, as well as pregabalin, was not effective in reducing pain and movements. Duloxetine (20 mg/day), in addition to pregabalin, produced partial pain relief and reduced movements. 

## Discussion

Although the causes of PLMT are varied, clinical factors associated with the disease have been noted. PLMT is more common in females (66%) with the age of onset ranging from 24 to 86 years [3]. The risk factors for PLMT include peripheral neuropathy, previous trauma, and radiculopathy, but cases are often idiopathic [3]. Although the present case had MRI findings of mild radiculopathy (L5), this finding is common in the general elderly population. Thus, the causality was challenging to establish in the present case. Pseudoathetosis is an important differential diagnosis in our case. However, the somatosensory system including deep sensation was normal, along with normal findings in the dorsal nerve in spinal MRI. Thus, pseudoathetosis was negligible in our case. It also should be noted that some cases with PLMT reportedly had a history of malignant tumors [3]. Thus, work-up for malignancy is critical in patients with PLMT (it showed negative findings in our case). 

The involuntary movements in PLMT were persistent and there was a gradual increase and decrease in the degree of symptoms. Even if the onset of symptoms were unilateral, more than half of the patients eventually developed bilaterally. Additionally, the patients were able to stop their involuntary movements transiently by intention or by pressing on the sole of the foot [4]. The involuntary movements seen in PLMT are unique and qualitatively different from conventional involuntary movements (tremor, myoclonus, and spasm) [5]. Thus, clinicians may easily mistake this condition for a psychological condition. However, the involuntary movements seen in PLMT are sharply different from the involuntary movements commonly observed in psychogenic disorders (e.g., posttraumatic dystonia) [6]. More specifically, there were no features of psychogenic movements in our case, such as 1) non-stereotypic symptoms, 2) symptoms increase with attention and decrease with distraction, 3) accompanied with pseudo muscle weakness, and 4) multiple distribution and pattern of movements [7].

Previous studies demonstrated that both pain and involuntary movements were refractory to medications [3,8]. Involuntary movements usually correlate with pain intensity in PLMT [9]. Treatments for pain rather than movement gave the present case a degree of relief, as seen in another study [3,10]. Given this discrepancy, it is likely that several pathological mechanisms seem to be involved in PLMT.

## Conclusions

Here, we report an elderly woman who was diagnosed with PLMT. Video shows typical unique movements, while multiple neurological examinations showed unremarkable findings. Chronic toe discomfort accompanied by involuntary movements might be caused by aching PLMT. If a patient suffers from the movements, multiple investigations should be done as described in this report. Additionally, clinicians should be aware of this unique movement disorder in order to avoid misdiagnosis with psychogenic conditions.
